# Data on community structure and diversity of the intestinal bacteria in elver and fingerling stages of wild Indonesian shortfin eel (*Anguilla bicolor bicolor*)

**DOI:** 10.1016/j.dib.2020.105299

**Published:** 2020-02-17

**Authors:** Diah Kusumawaty, Hertien Koosbandiah Surtikanti, Hernawati, Trina Ekawati Tallei

**Affiliations:** a Department of Biology Education, Faculty of Mathematics and Science Education, Universitas Pendidikan Indonesia, Bandung, Indonesia; b Department of Biology, Faculty of Mathematics and Natural Sciences, Universitas Sam Ratulangi, Manado, Indonesia

**Keywords:** *Anguilla bicolor bicolor*, Elver, Fingerling, Intestine, Metagenomic, Shortfin eel

## Abstract

This article describes the data on community structure and diversity of intestinal bacteria of Indonesian shortfin wild eel fingerling and elver (*Anguilla bicolor bicolor*). The specimens were obtained from Ci Kangean River, Alur Village, Cipatujah District, Tasikmalaya, West Java, Indonesia. The data were generated using DNA metagenomic approach on an Illumina paired-end platform by utilizing the V3–V4 region of the 16S rRNA gene. A total of 151,636 reads with 91.60% effective tags and 155,388 with 84.86% effective tags were generated from the intestine of wild eel fingerling (WF) and elver (WE), respectively. The total OTUs was 100 in WF and 358 in WE. The phyla Bacteroidetes (50%), Firmicutes (15%), Proteobacteria (13%), Fusobacteria (11%) and Verrucomicrobia (8%) were found in WF, and Proteobacteria (64%), Firmicutes (34%) and Fusobacteria (1%) were found in WE. The predominating families in WF were Porphyromonadaceae (50%), Clostridiaceae (12%), Fusobacteriaceae (10%), Verrucomicrobiaceae (8%), and in WE were Enterobacteriaceae (38%), Clostridiaceae (30%), Aeromonadaceae (17%), Moraxellaceae (7%). The predominating genera in WF were unassigned (48%), *Cetobacterium* (10%), *Clostridium* (sensu stricto) (9%), *Akkermansia* (8%), *Odoribacter* (4%), *Bacteroides* (4%), *Desulfovibrio* (4%), and in WE were *Plesiomonas* (36%), *Clostridium* (sensu stricto 1) (31%), *Aeromonas* (17%), *Acinetobacter* (7%). The amount of lactic acid bacteria found in the intestine of WF was 0.0028% and WE was 0.1218%. The data provide baseline information on the changes in the community and bacterial composition in line with the stages of growth and development of wild Indonesian shortfin eels.

**Table:** Specifications Table

Subject	Genetics, Genomics and Molecular Biology
Specific subject area	Metagenomics
Type of data	Table Chart Graph Figure
How data were acquired	Bacterial metagenomic was obtained by a paired-end platform using the V3–V4 region of the 16S rRNA gene.
Data format	Raw (https://data.mendeley.com/datasets/nb85yfr8my/1) Analyzed Filtered
Parameters for data collection	The total bacterial DNA isolated from the intestine of Indonesian shortfin wild eel fingerling and elver was extracted and sequenced using the V3–V4 region of the 16S rRNA gene on an Illumina paired-end platform
Description of data collection	The filtered sequence reads were analysed using bioinformatics pipeline.
Data source location	The specimens were obtained from Ci Kangean River, Alur Village, Cipatujah Ditrict, Tasik Malaya, West Java, Indonesia (coordinate −7.50545652, 107.82354261).
Data accessibility	Summarized data are provided within the article. Raw data are hosted in a public repository. Repository name: Mendeley data Direct URL to data: https://data.mendeley.com/datasets/nb85yfr8my/1

**Table:** 

**Value of the Data** • This is the first metagenomic profiling report on the intestinal bacterial diversity of wild elver and fingerling stages of an Indonesian shortfin eel (*Anguilla bicolor bicolor*) by a paired-end platform using the V3–V4 region of the 16S rRNA gene. • The data provide valuable information on the bacterial community structure of in the intestine of wild elver and fingerling stages of Indonesian shortfin eel (*A. bicolor bicolor*). • The data provide basic information about the different bacterial composition in the intestine of wild elver and fingerling stages of Indonesian shortfin eel (*A. bicolor bicolor*), which can be useful in the preparation of feed suitable for cultivated shortfin eels.

## 1 Data description

The data presented here are intended to provide information on the diversity of bacteria residing in the intestine of the elver and fingerling stages of wild Indonesian shortfin eel (*A. bicolor bicolor*). The data are very useful for predicting and assuming the balance of bacterial population in both specimens. In addition, the data are also important for the identification of the presence of *Aeromonas hydrophila*, a Gram-negative bacterium, which is a cause of death of fish found in warm aquatic environments [1]. The PCR-generated amplicons of the V3–V4 region of 16S rRNA genes were sequenced on an Illumina paired-end platform to generate 250 bp paired-end raw reads, and then assembled and pre-treated to obtain clean tags. Chimeric sequences in clean tags were detected and removed to finally obtain the effective tags. A total of 151,636 reads with 91.60% effective tags and 155,388 with 84.86% effective tags were generated from the intestine of wild eel fingerling (WF) and elver (WE), respectively. In order to analyze the species diversity in each sample, all effective tags were grouped by 97% DNA sequence similarity into OTUs (Operational Taxonomic Units) [2]. The total OTUs was 100 in WF and 358 in WE.

**Fig. 1:**
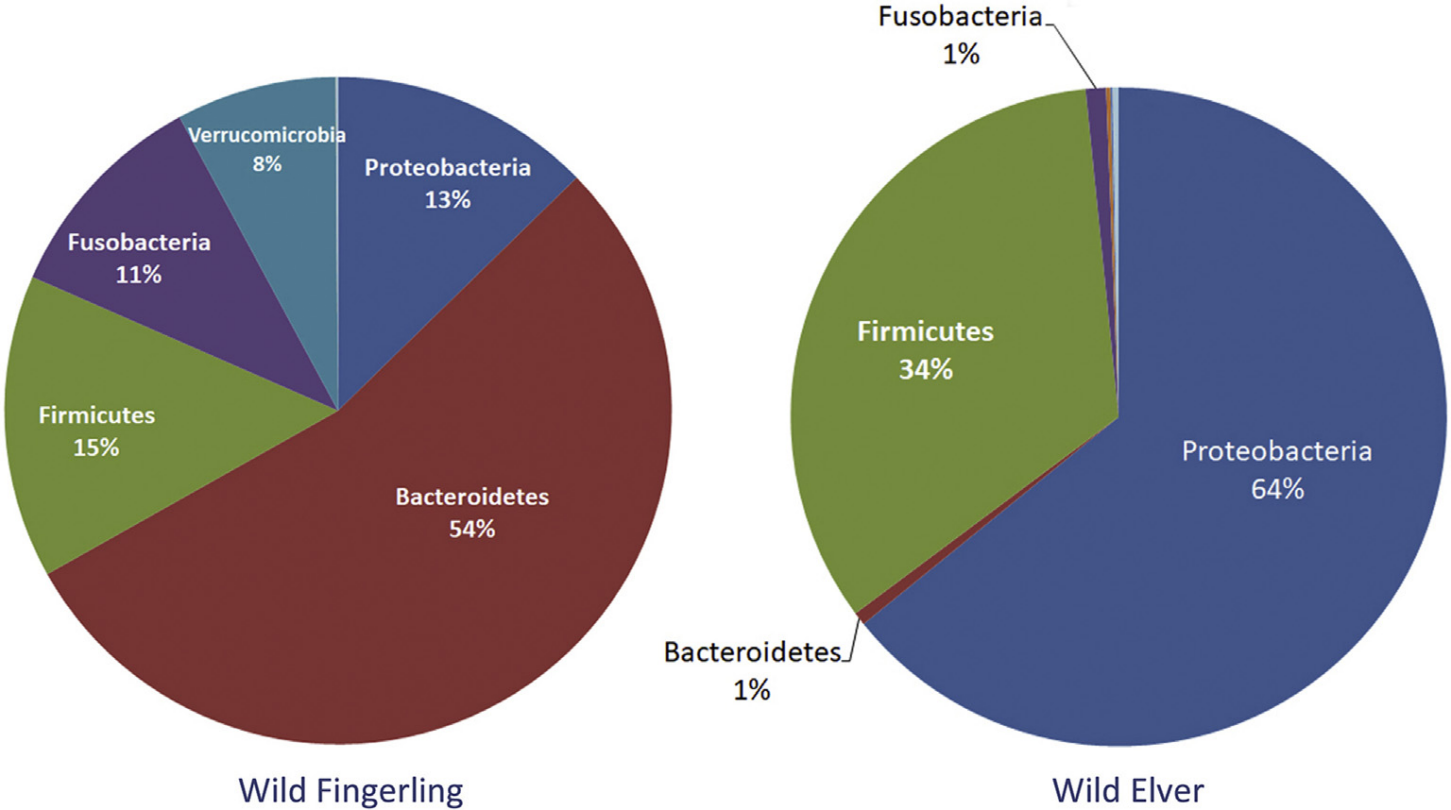
The relative abundance of phyla in both samples.

The relative abundance of the phyla in the intestines of WF and WE is presented in Table 1 and Fig. 1. A total of 9 phyla and 1 unassigned were found in WF, while 14 phyla and 1 unassigned were found in WE. Bacteroidetes (54%), Firmicutes (15%), Proteobacteria (13%), Fusobacteria (12%) and Verrucomicrobia (8%) appeared to be abundant in WF, while Proteobacteria (64%) and Firmicutes (34%) were abundant in WE.

**Table 1:** Relative abundance of phyla in the intestines of wild eel fingerling and elver.

Taxonomy	Wild Eel Fingerling (WF)	Wild Eel Elver (WE)
Proteobacteria	0.13	0.64
Bacteroidetes	0.54	0.01
Firmicutes	0.15	0.34
Fusobacteria	0.11	0.01
Verrucomicrobia	0.08	0.00002
Actinobacteria	0.00006	0.002
Cyanobacteria	0.000008	0.001
Gemmatimonadetes	0.00002	0.00005
Chloroflexi	0	0.00003
Tenericutes	0	0.0007
Saccharibacteria	0	0.00002
Spirochaetes	0.0001	0
Thermomicrobia	0	0.00002
Nitrospirae	0	0.00003
Elusimicrobia	0	0,00004
Others	0.0008	0.003

**Fig. 2:**
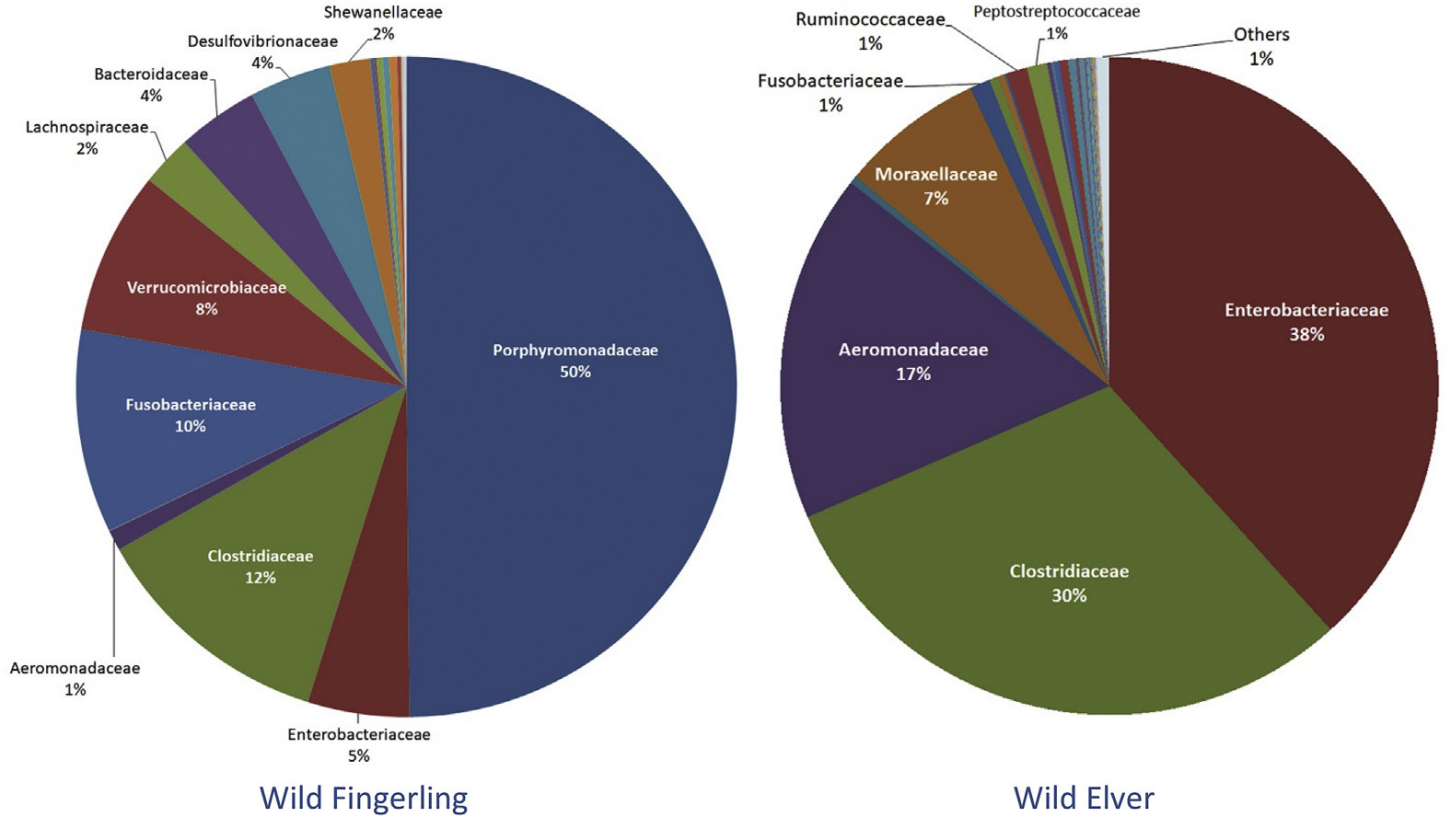
The relative abundance of family in both samples.

The family abundance in both samples is shown in Fig. 2. Porphyromonadaceae (50%), Clostridiaceae (12%), Fusobacteriaceae (10%), Verrucomicrobiaceae (8%), and Enterobacteriaceae (38%), Clostridiaceae (30%), Aeromonadaceae (17%), Moraxellaceae (7%) were predominating families in WF and WE, respectively.

**Fig. 3:**
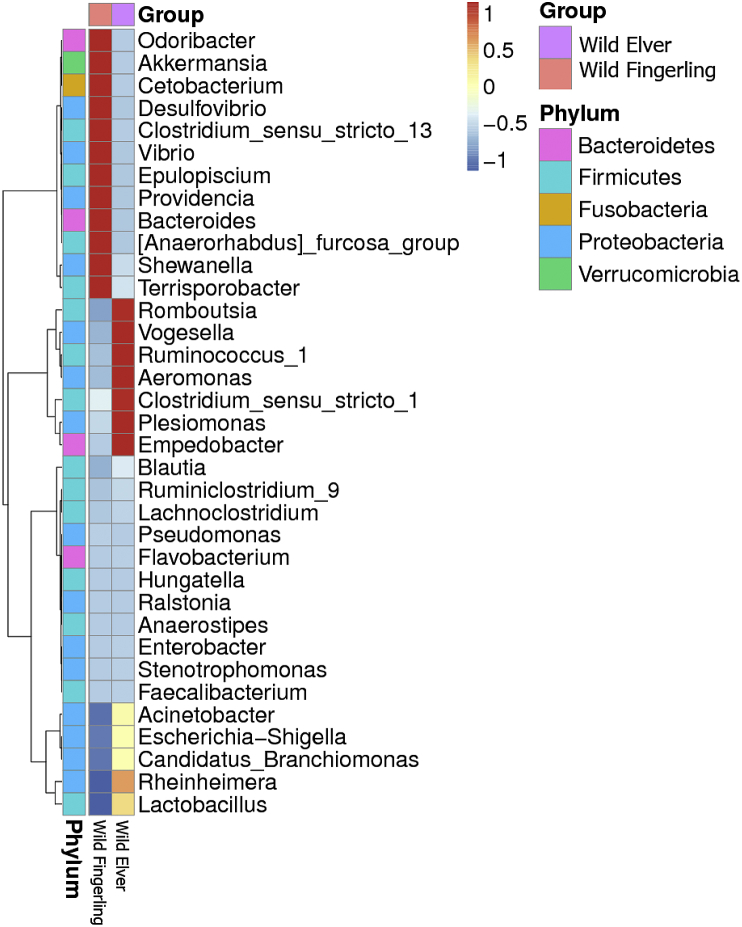
Heatmap representing the relative abundance of the genus level.

**Fig. 4:**
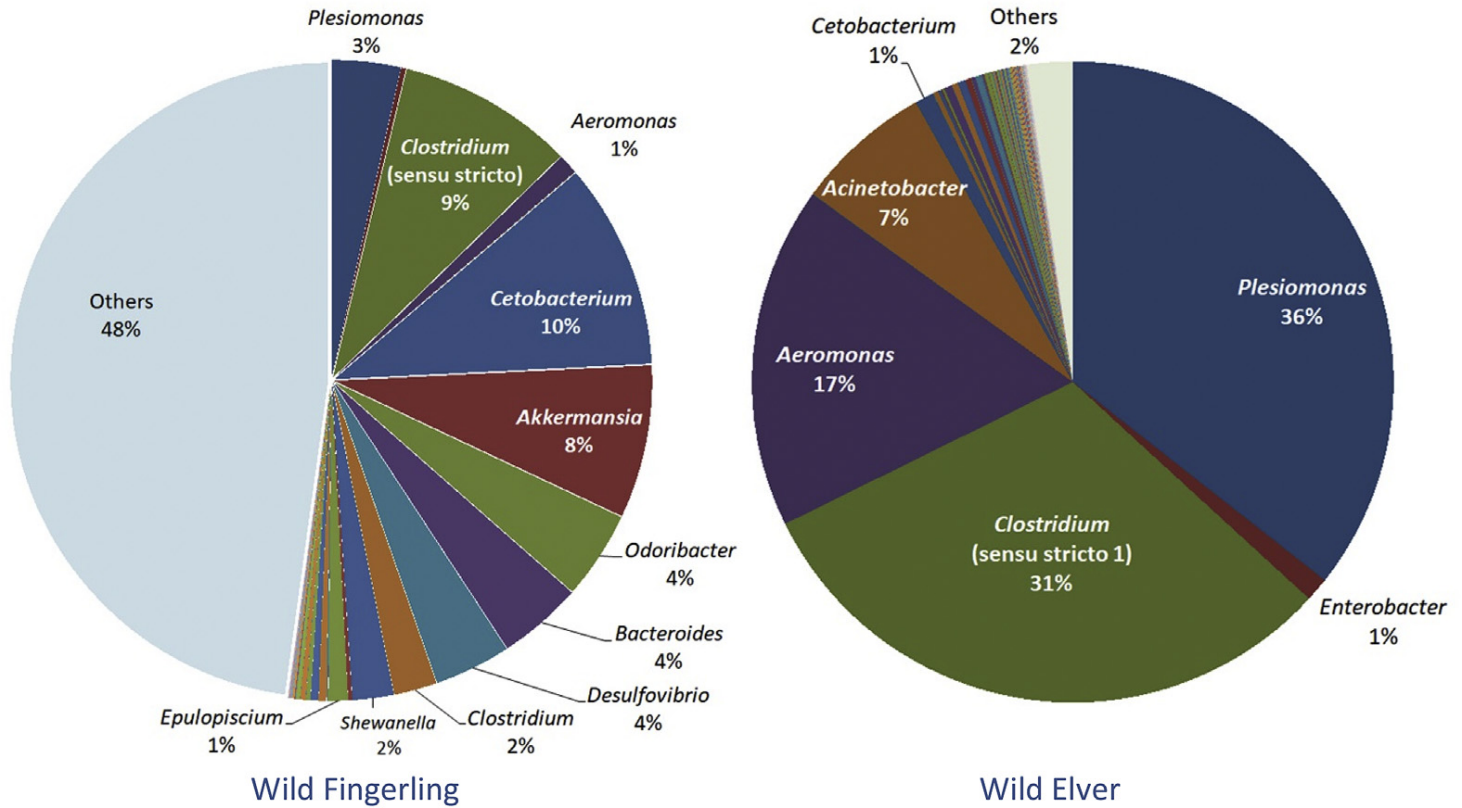
The most relative abundance of genera in wild eel fingerling and elver.

Relative taxonomic abundance heatmap of the genus level is shown in Fig. 3, while Fig. 4 shows the most relative abundance of genera in both specimens. Unassigned (48%), *Cetobacterium* (10%), and *Clostridium* (sensu stricto) (9%) were the most abundance genera in WF, while *Plesiomonas* (36%), *Clostridium* (sensu stricto 1) (31%), *Aeromonas* (17%), and *Acinetobacter* (7%) appeared to be predominating in WE.

**Fig. 5:**
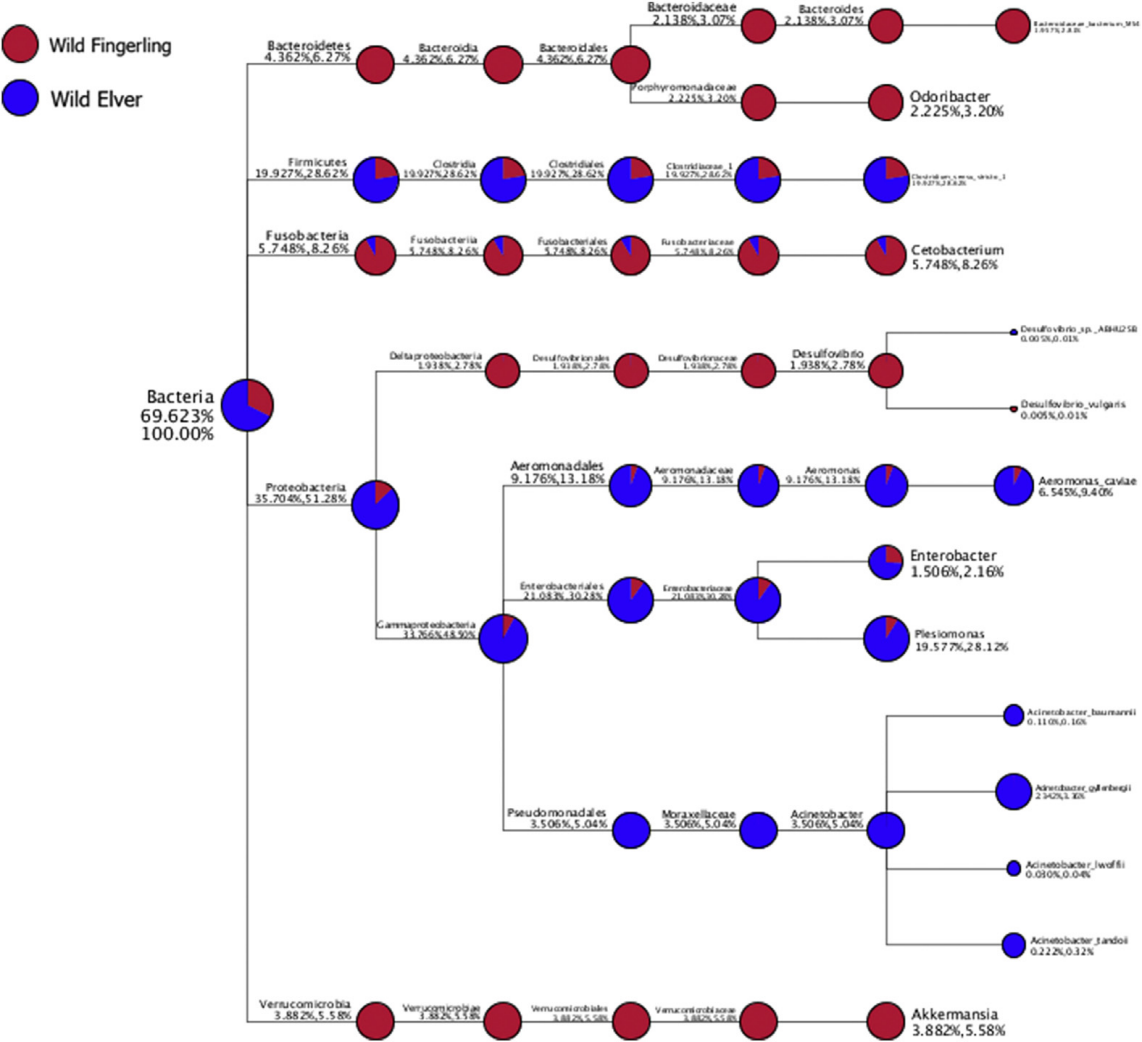
The hierarchical classification of the selected 10 genera/species with the high relative abundance in each intestine of WF and WE.

The top 10 genera/species with the high relative abundance were selected to generate a combined taxonomy tree in each sample (Fig. 5). The relative abundance of the whole and selected corresponding taxa were written as the first and the second numbers above the lines, respectively. Each taxonomic rank was colored differently. The relative abundance of the species was marked by the size of the circles. The percentage of the whole taxa and selected taxa were written below the lines.

Some very small amount of lactic acid bacteria was also detected and is shown in Table 2. The statistical indices of alpha diversity to assess microbial diversity within the community based on the clustering threshold 97% are summarized in Table 3.

**Table 2:** The list of lactic acid bacteria found in the intestine of wild eel fingerling and elver.

Species	Percentage (%)	
	Wild Eel Fingerling	Wild Eel Elver
*Lactobacillus murinus*	0	0.005
*L. intestinalis*	0.0008	0.06
*L. reuteri*	0	0.01
*L. jensenii*	0.002	0.01
*L. paralimentarius*	0	0.003
*L. fermentum*	0	0.0008
*Bifidobacterium animalis*	0	0.03
*B. dentium*	0	0.003
Total	0.0028	0.1218

**Table 3:** Alpha diversity indices of species.

Samples	Observed Species	Shannon	Simpson	Chao1
Wild fingerling	100	3.65	0.86	115,111
Wild elver	358	2.89	0.73	358,038

**Fig. 6:**
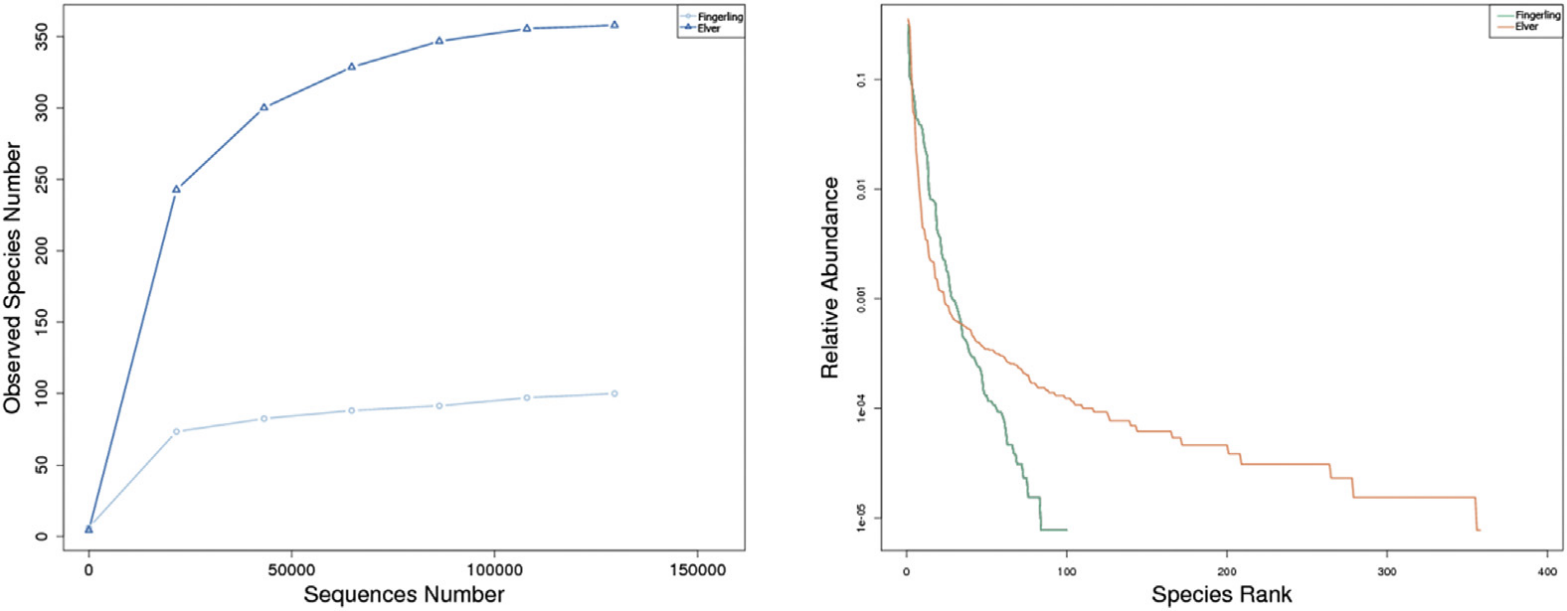
(a) Rarefaction curves and (b) Rank abundance curves. In (a), the dark blue color is the sample from WF, and the light blue color is the sample from WE. In (b), the green color in (b) is the sample from WF, and the red color is the sample from WE.

Rarefaction and Rank abundance curves are shown in Fig. 6. These curves are used extensively to identify biodiversity in samples. The curve has reached the plateau so it is said that a large number of bacterial species have been identified. The abundance curve rank displays the relative abundance of species and visualizes species richness and evenness.

**Fig. 7:**
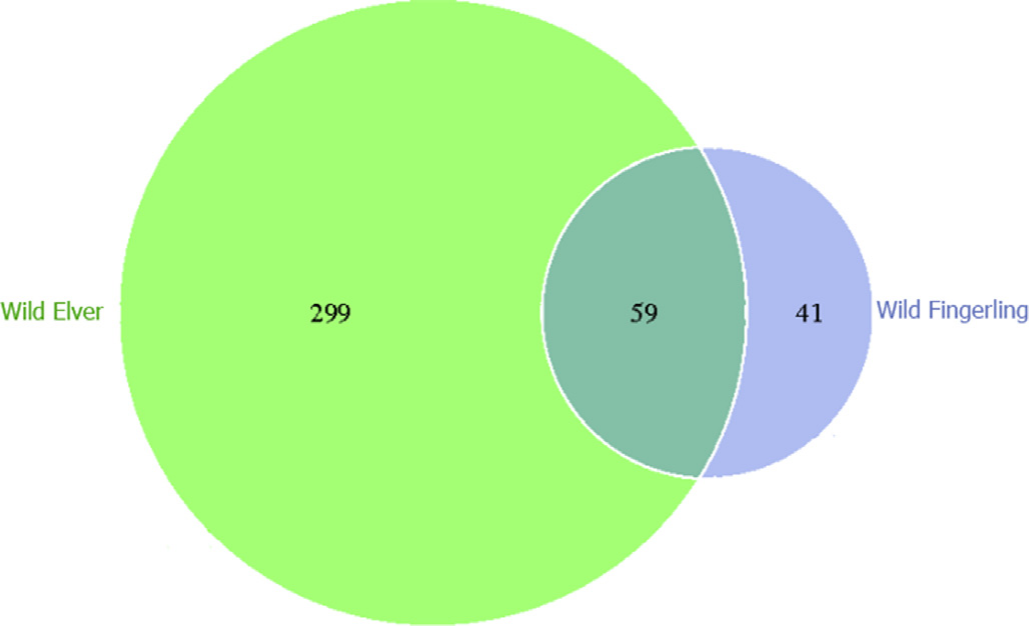
Venn diagram based on OTUs.

The Venn diagram based on OTUs generated after being normalized and the common and unique information from different samples were analyzed is shown in Fig. 7. The diagram shows that there are 59 common species shared by the two samples.

## 2 Experimental design, materials, and methods

### 2.1 Sample preparation

The Indonesian wild eel fingerling and elver were obtained from Ci Kangean River, Alur Village, Cipatujah District, Tasikmalaya, West Java, Indonesia (coordinate −7.50545652, 107.82354261). The procedure for isolation of the intestine followed Huang et al. [3] with modification. The samples were euthanized, surface-sterilized with 70% ethanol for 90 s, and then rinsed three times with sterilized deionized water. The intestine was cut out using a sterile scissor and placed immediately in a sterile 50-ml tube containing sterile PBS. It took 16 individuals of WE and 3 individuals of WF to obtain each 0.5 g of intestines.

### 2.2 Extraction of genomic DNA

The preparation of the samples' genomic DNA was done using CTAB/SDS method. The concentration and purity of DNA were analyzed on 1% agarose gels. Based on the concentration, DNA was diluted to 1ng/μL in sterile water.

### 2.3 The generation of amplicons

The V3–V4 regions of 16S rRNA were amplified using Phusion® High-Fidelity PCR Master Mix (New England Biolabs). The PCR products were mixed with the same volume of 1x loading buffer and electrophoresed on 2% agarose gel. Samples with bright and sharp bands between 400-450 bp were chosen for further experiments.

### 2.4 PCR products mixing and purification

PCR products are mixed in a balanced ratio then purified with Qiagen Gel Extraction Kit (Qiagen, Germany). The library was generated with NEBNext® UltraTM DNA Library Prep Kit for Illumina, quantified via Qubit and Q-PCR, and analysed by Illumina platform.

### 2.5 Bioinformatics analysis

#### 2.5.1 Sequencing data processing

The barcodes of the samples were cut off from their primer sequences, assigned as pair-end reads and merged using FLASH (V1.2.7) [4] to produce raw tags. Quality filtering was conducted to obtain high-quality clean tags [5] according to the Qiime V1.7.0 [6] quality control process. The tags were compared with the reference database (Gold database) using UCHIME algorithm [7] to detect chimera sequences. Finally, the chimera sequences were removed [8] to obtain the Effective Tags.

#### 2.5.2 OTU cluster and species annotation

Uparse software v7.0.1001 [9] was used for sequence analysis using all the effective tags. Sequences with ≥97% similarity were assigned to the same OTUs. Further annotation was assigned to a representative sequence for each OTU after being screened. For each representative sequence, Mothur software was performed against the SSUrRNA database (SILVA Database) [10] for species annotation at each taxonomic rank with Threshold 0.8 [11]. To get the phylogenetic relationship of all OTUs representative sequences, MUSCLE V. 3.8.31 was used to compare multiple sequences. Normalization of OTUs abundance information was performed using a standard of sequence number that corresponds to the sample with the least sequences. Subsequently, analysis of alpha diversity was conducted on these normalized data.

#### 2.5.3 Alpha diversity

QIIME V. 1.7.0 was used to generate the alpha diversity for species to measure the average mean of taxa diversity in both samples. The alpha diversity curve was displayed with R software V. 2.15.3.
